# Short term survival of critically ill COVID-19 Egyptian patients on assisted ventilation treated by either Dexamethasone or Tocilizumab

**DOI:** 10.1038/s41598-021-88086-x

**Published:** 2021-04-23

**Authors:** Alaa Rashad, Sherif Mousa, Hanaa Nafady-Hego, Asmaa Nafady, Hamed Elgendy

**Affiliations:** 1 Department of Chest Diseases and Tuberculosis, Qena Faculty of Medicine, South Vally University, Qena, Egypt; 2 Esna Quarantine Hospital, Esna, Luxor Egypt; 3 Department of Microbiology and Immunology, Faculty of Medicine, Assiut University, Assiut, Egypt; 4 Department of Clinical Pathology, Faculty of Medicine, Assiut University, Assiut, Egypt; 5 Department of Clinical and Chemical Pathology, Qena Faculty of Medicine, South Valley University, Qena, Egypt; 6 Department of Anaesthesia, Faculty of Medicine, Assiut University, Assiut, Egypt; 7 Anaesthesia Department, HAMAD Medical Corporation, Doha, Qatar

**Keywords:** Diseases, Medical research

## Abstract

Tocilizumab (TCZ) and Dexamethasone are used for the treatment of critically ill COVID-19 patients. We compared the short-term survival of critically ill COVID-19 patients treated with either TCZ or Dexamethasone. 109 critically ill COVID-19 patients randomly assigned to either TCZ therapy (46 patients) or pulse Dexamethasone therapy (63 patients). Age, sex, neutrophil/ lymphocyte ratio, D-dimer, ferritin level, and CT chest pattern were comparable between groups. Kaplan–Meier survival analysis showed better survival in Dexamethasone group compared with TCZ (*P* = 0.002), patients didn’t need vasopressor at admission (*P* < 0.0001), patients on non-invasive ventilation compared to patients on mechanical ventilation (*P<0.0001* ), and in patients with ground glass pattern in CT chest (*P<0.0001* ) compared with those who have consolidation. Cox regression analysis showed that, TCZ therapy (HR = 2.162, 95% CI, 1.144–4.087, *P* <0.0001) compared with Dexamethasone group, higher neutrophil/Lymphocyte ratio (HR = 2.40, CI, 1.351–4.185, *P* = 0.003), lower PaO_2_/FiO_2_, 2 days after treatment, (HR = 1.147, 95% CI, 1.002–1.624, *P* < 0.0001) independently predicted higher probability of mortality. Dexamethasone showed better survival in severe COVID-19 compared to TCZ. Considering the risk factors mentioned here is crucial when dealing with severe COVID-19 cases.

*Clinical trial registration No* clinicalTrials.gov: Nal protocol approved by Hospital Authorities, for data collection and for participation in CT04519385 (19/08/2020).

## Introduction

After the worldwide spread of SARSCov-2 infection, an intense need for therapeutics to treat critically ill patients in ICU is mandated. In addition to the supportive treatment several regimens have been tremendously used to manage critically ill COVID-19 patients^1,2^. There is a controversy regarding the use of glucocorticoids in treating critically ill COVID-19 patients. Proponents of its use cite its ability to suppress the inflammation of the lung^3^, while opponents cite its immune suppressive effect that can precipitate secondary bacterial infection and worsen the patients’ outcome^4^. Tocilizumab (TCZ) was used in COVID-19 to control cytokine storm and lung inflammation^5^. Furthermore, one study used glucocorticoids with or without TCZ to manage patients with COVID-19^3^.

Identification of the factors associated with mortality is crucial to set the proper management, the Chinese researchers found ground-glass opacities mixed with consolidations were associated with mortality of patients infected with coronavirus in China and invented a single computed tomography score to predict mortality^6^. Male gender, elderly and presence of comorbidities were associate with death in patients with COVID-19^7^, hence clinical and laboratory investigations should be thoroughly done for COVID-19 patients^8^.

The primary objective of our study is to study the patients’ survival after treatment with either (TCZ) or dexamethasone in critically- ill patients with COVID-19 in the intensive care unit (ICU) setting. The secondary objective is to examine the association of radiological findings, and the partial pressure of arterial oxygen over the fraction of inspired oxygen (PaO_2_/FiO_2_ ratio), on the outcome of these critically ill patients.

## Study design and patients

The current study takes place in the ICU of ESNA hospital, the first COVID-19 quarantine hospital in Egypt, located in Upper Egypt, Luxor governorate during the period from March 2020 to June 2020.

The diagnosis of COVID-19 was confirmed with a positive RT-PCR results.PCR testing was performed on aliquots of Universal Transport Medium (UTM) used for nasopharyngeal swabs’ collection (Liuyang Sanli Medical Technology Development China). Aliquots were: extracted on the QIA symphony platform (QIAGEN, USA) and tested with real-time reverse-transcription PCR (RT-qPCR) using the QIAamp DSP Virus Spin Kit (QIAGEN Hilden, Germany) on a Rotor-Gene Q (QIAGEN Hilden, Germany). Patients data were collected from the medical records at day one of treatment initialization and included patients' demographics, comorbidities, symptoms, oxygen support, a ratio of the partial pressure of arterial oxygen over the fraction of inspired oxygen (PaO_2_/FiO_2_ ratio), laboratory values (complete blood count, CRP, D-dimer, ferritin, AST, ALT), diagnostic workup, therapies, complications, and outcomes, CT chest either mainly ground glass infiltrated or combined ground glass and consolidation^9^, PaO_2_/FiO_2_ ratio data were also collected after two days from the end of the treatment and overall mortality within 14 days.

All patients provided informed consent for treatment according to the local protocol approved by Hospital Authorities, for data collection and for participation in the study. The study was carried out under the principles of the Declaration of Helsinki and approved by the Regional Ethics Committee, Qena faculty of medicine, South-Valley University (SVUMEDCH019420862).

Eligibility for study included patients with significant deterioration in respiratory clinical status with respiratory rate > 30 cycle/minute, Bilateral Chest computed tomography (CT) infiltration > 30%, PaO_2_/FiO_2_ ratio < 150 or saturation < 90 on > 6 L/min, Two positive laboratory tests of the following: (CRP > 10 g/L, lymphocytes < 600 /mm3, D-dimer > 500 ng/mL , ferritin > 500 ng/mL). Interleukin-6 levels were not part of the criteria because it did not necessarily result in an actionable time frame^10^.

Regarding vasopressors, our protocol has started with norepinephrine and add vasopressin if the patient didn’t respond, echocardiography is mandatory to rule out pulmonary embolism (PE) or myocardial dysfunction in unresponsive patients.

Regarding anticoagulation, all our patients in ICU received enoxaparin 1 mg/Kg subcutaneous twice daily, if creatinine clearance < 30 ml/min dalteparin 100 units/Kg subcutaneous twice daily.

Pediatric patients < 18 years old, patients with an active bacterial or fungal infection, patients on chemotherapy, patients with interstitial lung disease and patients who were not requiring supplemental oxygen were excluded from the study.

The endpoint was time to failure, defined as death, within 14 days from ICU admission. Patients with a very early death reduced the risk that the treatment choice was motivated by the patient's disease course. Therefore, day three from hospital admission was set as a landmark time point: those who died before the 3rd day of ICU admission were excluded^11^.

## Randomization and masking

Patients were randomly assigned (1:1) to Dexamethasone therapy plus standard of care or TCZ plus standard of care. Randomization in blocks of variable size was performed in an electronic case report form system and stratified by site, age (≥ 50 years versus < 50 years), baseline PaO_2_/FiO_2_, and respiratory status (non-invasive positive-pressure ventilation, or mechanical ventilation).

The study didn't comprise a placebo-comparator group since this is considered inappropriate in an era of a pandemic with substantial global mortality and treating critically ill patients on assisted ventilation**.**

## Procedure

Patients were given symptomatic treatment for COVID-19, in addition to azithromycin 500 mg/day for 7 days, Oxygen therapy and non-invasive or mechanical ventilation when needed. In addition to conventional treatment, patients were randomly assigned to either of Dexamethasone group or Tocilizumab (TCZ) group.

### The Dexamethasone group

Received pulse Dexamethasone 4 mg/kg/day in an infusion form for 3 days, followed by a maintenance dose of 8 mg/day for ten days.

### The Tocilizumab group

Received TCZ, 4 mg/kg/dose in 100 cc normal saline over one hour repeated after 24 h, then patient continue symptomatic treatment and oxygen therapy and/or assisted ventilation as needed.

Patients, investigators, and health-care providers were not masked to study drug assignment.

### Statistical analysis

Statistical Package of Social Sciences (IBM-SPSS 21), (SPSS: IBM Company, version 21.0, IBM Corporation, Armonk, NY, USA) was used for data analysis. Normal distribution of data was assessed by Shapiro–Wilk’s test (non parametric tests were used on *P* < 0.05). Mann–Whitney U test was used to compare variables between the studied groups. Chi-square test were used to compare ratio difference between the studied groups. Wilcoxon's test was used to compare PaO_2_/FiO_2_ before and after tocilizumab administration). Statistical significance was set at *P* < 0.05.

Quantitative data presented as median and IQR (interquartile range)and qualitative data presented in the form of frequency and percentage.

Kaplan–Meier survival curves were constructed to show cumulative mortality over the 14 days, Cox proportional hazards analysis was performed to test for association between baseline characteristics and survival, with censoring of data on day 14 for patients who had died during hospitalization.

## Results

**Figure 1:**
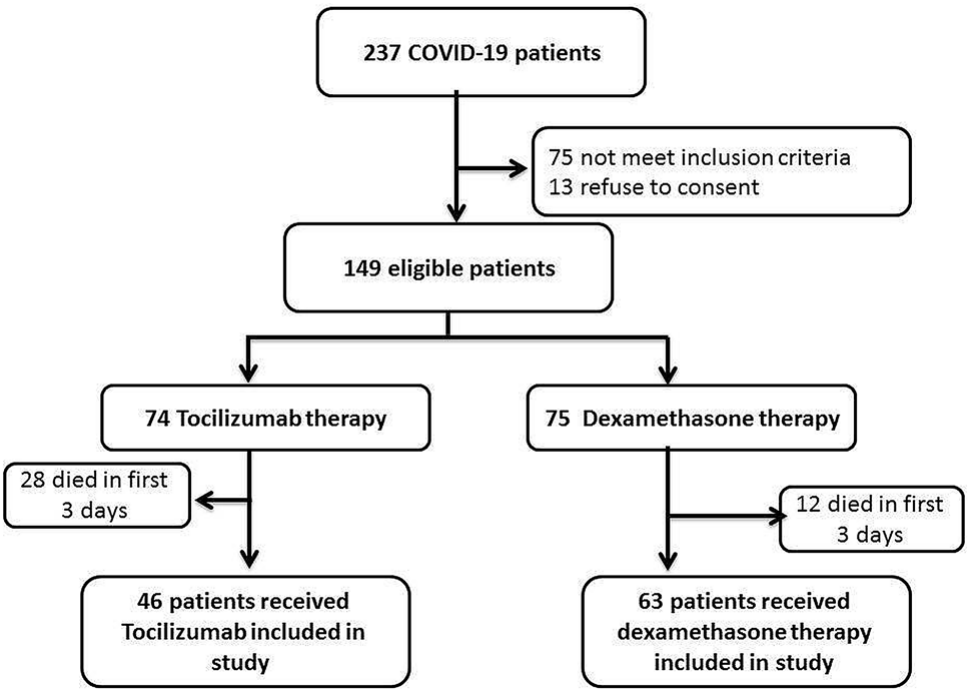
Study flow chart.

A total of 237 patients were admitted to intensive care unit during the study period, 75 patients didn’t meet inclusion criteria, 13 patients refused to consent, the remaining 149 were randomly assigned to either Dexamethasone or tocilizumab group; out of 74 patients in the Dexamethasone group, 12 patients died in the first 3 days of admission, the remaining 63 patients were included in the study as Dexamethasone group, on the other hand, out of 75 patients in TCZ group, 28 patients died in the first 3 days of admission the remaining 46 patients were included as TCZ group (Fig. 1).

The clinical features, comorbidities, total number of deaths, Complications and laboratory data of patients enrolled in this study were shown in Table 1. According to the medication given, patients are stratified into two groups. The first group contains 46 patients; all of them received TCZ therapy. In comparison, the second group includes 63 patients, all received Dexamethasone therapy, and the two groups were of comparable age and gender. The median age (IQR [interquartile range]) was 60 years (IQR 49.5, 66.5) for the TCZ group and 64 years (IQR 55, 72) for the Dexamethasone group, *P* = 0.056. The TCZ group included 26 men and 20 women, while the Dexamethasone group included 36 men and 27 women *p* = 0.948.

**Table 1:** Showed clinical characteristics of studied patients with COVID-19 under tocilizumab or pulse Dexamethasone therapy.

	COVID-19 patients under tocilizumab Therapy	COVID-19 patients under pulse Dexamethasone Therapy	*P* value
Number	46	63	
The median age (IQR [interquartile range]) / years	60.5 (49.5, 66.5)	64 (55, 72)	0.056
Sex (male / female)	(26/ 20)	(36/ 27)	0.948
PaO_2_/FiO_2_ before treatment median (IQR)	84 (75.75, 96.25)	82 (70, 90)	0.2
PaO_2_/FiO_2_ 2 days after treatment median (IQR)	82 .2 (78, 124)	92 (71, 129)	0.344
Neutrophiles/ Lymphocytes ratio median (IQR)	3.72 (3.62, 4.11)	3.69 (3.1, 3.69)	0.057
*D-dimer ng/mL* median (IQR)	1420 (420, 3720)	1400 (750, 3200)	0.897
Ferritin ng/mL median (IQR)	1153.5 (661, 1562.5)	934 (623, 1357)	0.124
NIV/ MV	32/14	38/25	0.32
CT Chest			
GGO/Consolidation	16/30	20/43	0.448
Vasopressor before treatment	28	27	0.081
*Comorbidities number (%)*			
Diabetes mellites	16 (34.8%)	15 (23.8%)	0.149
Hypertension	26 (56.5%)	26 (41.3%)	0.84
Heart diseases	0	14 (22.2%)	< 0.0001
Kidney disease	4 (8.7%)	5 (7.9%)	0.576
Cancer	0	1 (1.6%)	0.578
Hyperthyroidism	0	1 (1.6%)	0.578
hepatitis C virus	2 (4.3%)	2 (3.2%)	0.565
COPD	0	2 (3.2%)	0.332
Asthma	0	3 (4.8%)	0.189
Deaths number (%)	32 (69.6%)	33 (52.4%)	0.05
Nasocomial pneumonia	14 (30.4%)	16 (25.4%)	0.356
Hyper glycemia	6 (13%)	9 (14.3%)	0.542
Renal impairment	3 (6.5%)	2 (3.2%)	0.353
Increased liver enzymes	6 (13%)	5 (7.9%)	0.288
Pulmonary embolism	3 (6.5%)	1 (1.6%)	0.201

*Abbreviations* MV: mechanical ventilation; NIV: Non-invasive ventilation; PaO_2_/FiO_2_: ratio of partial pressure of arterial oxygen over fraction of inspired oxygen; *P* ˂ 0.05 considered statistically significant. GGO ground glass opacities.

**Figure 2:**
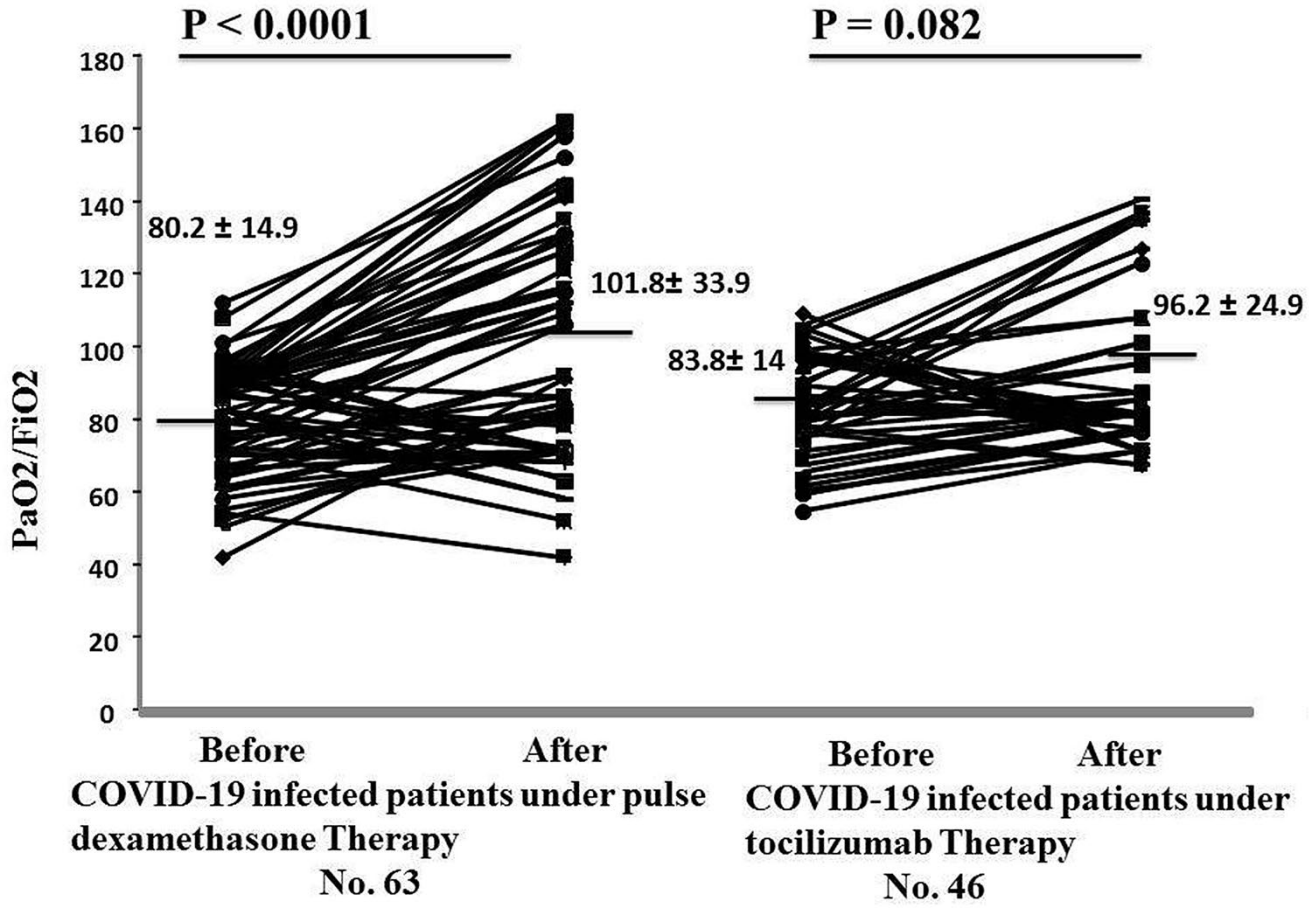
Showed PaO_2_/FiO_2_ variations after exposure of infected patients with COVID-19 to either tocilizumab or pulse Dexamethasone therapy. P ˂ 0.05 considered statistically significant.

Investigation of the improvement of PaO_2_/FiO_2_ ratio revealed that in the TCZ group the PaO_2_/FiO_2_ ratio, does not significantly improve after two days of TCZ therapy *P* = 0.082, while patients who received Dexamethasone Therapy their PaO_2_/FiO_2_ ratio significantly improved after two days therapy [TCZ group, PaO_2_/FiO_2_ ratio, Before treatment 83.8 ± 14 and after treatment 96.2 ± 33.9, *P* = 0.082) Dexamethasone therapy, PaO_2_/FiO_2_ ratio, Before treatment 80.2 ± 14.9 and after treatment 101.8 ± 33.9, *P* < 0.0001) (Fig. 2).

### Patients' outcome and survival analysis

We followed our patients for 14 days after admission to ICU. Baseline data included Neutrophil/ lymphocyte ratio, D-dimer, ferritin and CT chest pattern, were comparable between the 2 studied groups (TCZ group and Dexamethasone group), Neutrophil/ lymphocyte ratio 3.72 (3.62, 4.11) and 3.69 (3.1, 3.69) respectively, *P* = 0.057), D-dimer ng/mL1420 (420, 3720) and 1400 (750, 3200) respectively, *P* = 0.897), ferritin ng/mL1153.5 (661, 1562.5) and 934 (623, 1357) respectively, *P* = 0.124) before starting the treatment (Table 1).

**Figure 3:**
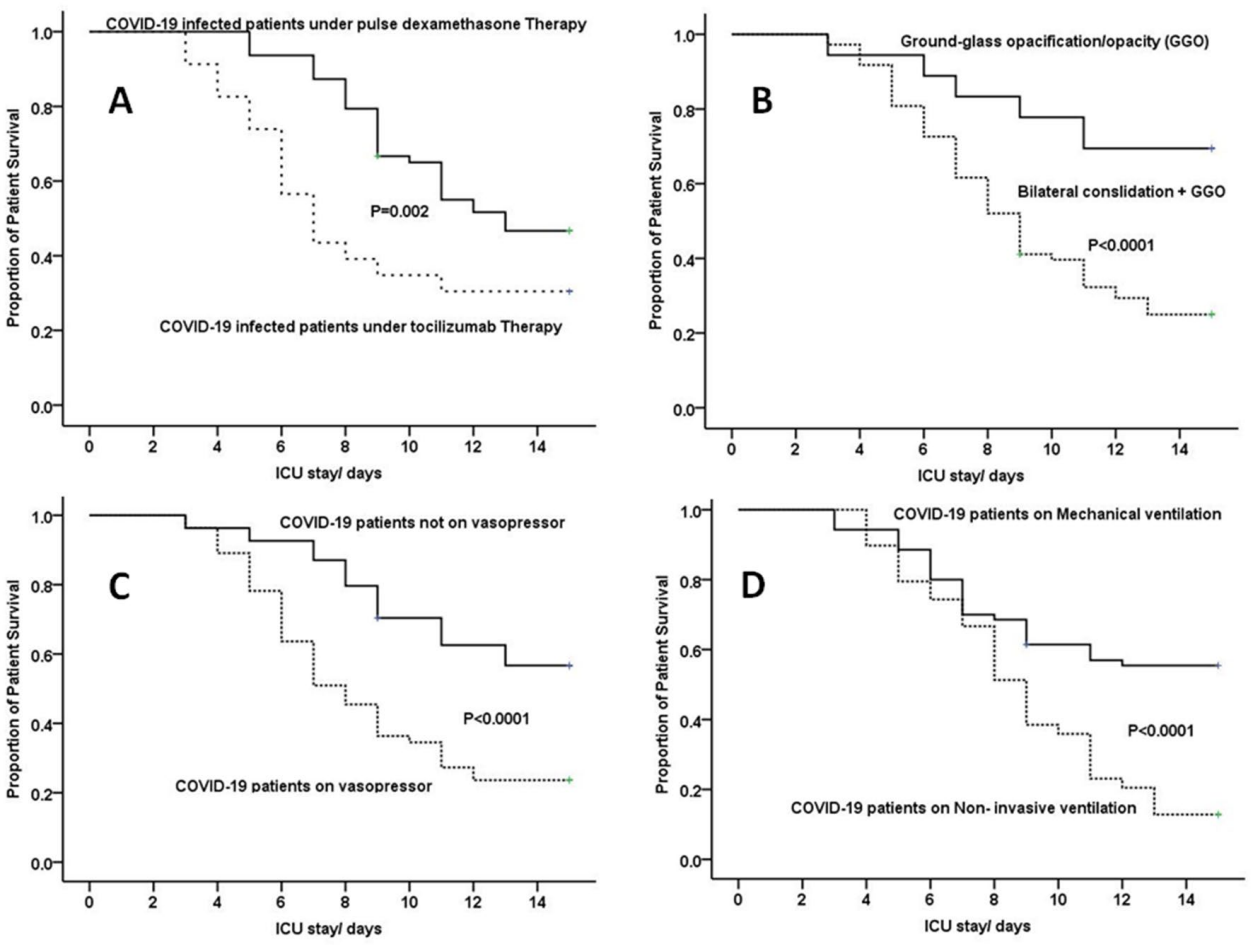
Showed Kaplan–Meier survival curve of critically ill COVID-19 patients, (**A**) for patients who received Toclizumab compared to those received pulse Dexamethasone (*P* = 0.002). (**B**) for patients with CT Chest showing Ground glass opacities (GGO) compared with bilateral consolidation, (*P* < 0.0001). (**C**) patients who do not need vasopressors at admission and those who need vasopressors at admission (*P* < 0.0001). (**D**) patients on mechanical ventilation compared to those on Non-invasive ventilation (*P* < 0.0001).

The presence of bilateral consolidation adding to ground-glass opacities (GGO) in CT scan significantly associated with a patient's survival, with worsen outcome in patients with bilateral consolidation, (*P* < 0.0001), (Fig. 3).

Interestingly, we found that patients who received Dexamethasone therapy showed better survival compared with those who received TCZ therapy *P* = 0.002) (Fig. 3).

Mechanical ventilation was associated with higher mortality compared to non-invasive ventilation (*P* < 0.0001), also need for vasopressors at time of admission was associated with mortality (*P* < 0.0001) (Fig. 3).

Risk factors associated with mortality in severe COVID-19 patients were addressed using Cox proportional hazards regression model cases, variables which revealed *P*value ≤ 0.1 in univariate analysis were included in multivariable analysis.

TCZ therapy (HR = 2.16, 95% CI, 1.14–4.09, P < 0.0001) compared with Dexamethasone group, higher neutrophil/Lymphocyte ratio (HR = 2.40, CI, 1.40–4.20, *P* = 0.003), lower PaO_2_/FiO_2_, 2 days after treatment, (HR = 1.15, 95% CI, 1.00–1.62, *P* < 0.0001) independently predicted higher probability of mortality (Table 2).

**Table 2:** Multivariable COX regression analysis of mortality in 109 critically ill COVID-19 patients.

Variable	HR	(95% CI)	P Value
Use of vasopressor	0.593	(0.593–0.327)	0.086
GGC/consolidation	1.104	(0.753–1.627)	0.659
Ferritin ng/mL	1.028	(1.001–1.076)	0.021
PaO_2_/FiO_2_before treatment	1.037	(1.029–1.002)	0.034
PaO_2_/FiO_2_, 2 days after treatment	1.147	(1.002- 1.624)	< 0.0001
Neutrophil/Lymphocyte ratio	2.4	(1.351- 4.185)	0.003
D-dimer ng/mL	0.965	(0.831–1.121)	0.640
TCZ/Dexamethasone	2.162	(1.144–4.087)	< 0.0001

Abbreviations GGC: ground glass opacities; COPD: Chronic obstructive pulmonary disease; PaO_2_/FiO_2_: ratio of partial pressure of arterial oxygen over fraction of inspired oxygen TCZ: Tocilizumab; *P* ˂ 0.05 considered statistically significant.

## Discussion

COVID-19 rapidly spread from China to all areas worldwide. The severe manifestation of severe acute respiratory syndrome coronavirus 2 (SARS-CoV-2), the causative agent of COVID-19, raised challenges for intensive care professionals to manage this syndrome. One of the most critical situations associated with COVID-19 is cytokine storms that can happen in up to 25% of the patients and found to be responsible for fatality ^12^. Herein, we compared the survival outcome of severe COVID-19 patients after treatment with either high dose Dexamethasone or TCZ, interestingly, we found that patients who received Dexamethasone therapy patients showed better survival when compared with those who received TCZ therapy, other factors that significantly associated with improved survival include, ground glass pattern in CT chest compared to consolidation were also included. Ramiro and his colleagues^3^ reported better improvement of respiratory recovery, patients' survival and reduced need for invasive mechanical ventilation in COVID-19-associated cytokine storm syndrome (CSS), after the endorsement of the regimen of high-dose glucocorticoids, then TCZ. However, their study design was a case–control study not randomized, in which they compared combined Dexamethasone and TCZ therapy with conventional therapy. Hence, their results cannot be compared with our results due to different methodology^12^.

Some case reports and one case series on the treatment with TCZ have been reported in the literature, suggesting some benefits in severely ill patients^3,5–8,13,14^. A previous descriptive study showed the proper use of TCZ in treating patients with cytokine storm features outside of the ICU setting and in non-ventilated patients. Again, the difference between their study methodology and ours challenges the comparison. They found that TCZ was associated with shorter vasopressor support; the shorter median duration of invasive ventilation and the shorter median time to clinical improvement and shorter hospital stay^15^.

In agreement with our results, Lan et al. performed a meta-analysis of TCZ for severe COVID-19^16^. They found that the use of TCZ for severe COVID-19 is not of great benefit and does not lead to an improvement in the term or reducing mortality, need for ICU admission, the requirement of mechanical ventilation, as they found similar mortality rates among severe COVID-19 regardless use of TCZ. TCZ and control groups were (16.2% [39/241] vs. 24.1% [85/352], RR, 0.61; 95% CI, 0.31–1.22. Besides, the risk of ICU admission was similar between the TCZ and control groups (35.0% vs. 15.8%, RR, 1.51; 95% CI, 0.33–6.78), the need of mechanical ventilation was similar between the TCZ and control groups (32.4% vs. 28.6%, RR, 0.72; 95% CI, 0.05–10.96, I2 = 91%)^17^.

Concerning dexamethasone efficacy in severe COVID 19, our study showed a significant survival benefit in patients treated with high dose dexamethasone, in agreement with results of the RECOVERY trial, which found that the use of dexamethasone resulted in lower 28-day mortality among patients who were receiving either invasive mechanical ventilation or oxygen alone at randomization but not among those receiving no respiratory support^18^.

Grasselli and his colleagues described variables risk factors for mortality for patients with COVID-19^19^. He included old age, male gender, and history of other chronic morbidities, for instance, hyperglycemia, hyperlipidemia, and chronic pulmonary diseases. Moreover, patients who need high oxygen support on admission were at higher risk^20^.

A recent study identified the most prevalent causes of mortality for those patients, first, was multiple organ failure, and followed by decompensated heart disorder and pulmonary system failure^21^.

In conclusion, our study investigated the efficacy of TCZ compared to pulse dexamethasone therapy in treatment of severe COVID-19 cases, and we found a significantly improved survival in patients who received pulse dexamethasone therapy.

### Informed consent

Institutional Review Board (IRB), Qena Faculty of Medicine, Qena, Egypt approved this study (SVUMEDCHT019420863). The study was conducted in accordance with the Declaration of Helsinki.
